# Striking sex differences in magnetic resonance imaging findings in the sacroiliac joints in the population

**DOI:** 10.1186/s13075-021-02712-7

**Published:** 2022-01-20

**Authors:** J. Braun, X. Baraliakos, R. Bülow, C. O. Schmidt, A. Richter

**Affiliations:** 1 Rheumazentrum Ruhrgebiet, Claudiusstr.45, 44649 Herne, Germany; 2 Ruhr University Bochum, Bochum, Germany; 3 Department of Diagnostic Radiology and Neuroradiology, University Medical Center Greifswald, Greifswald, Germany; 4 Institute for Community Medicine, University Medical Center Greifswald, Greifswald, Germany

**Keywords:** Axial spondyloarthritis, Magnetic resonance imaging, HLA-B27, Bone marrow edema, Machine learning

## Abstract

**Background:**

In patients with axial spondyloarthritis (axSpA), magnetic resonance imaging (MRI) is used to detect bone marrow edema (BME) in sacroiliac joints (SIJ) but SIJ BME are also detected in the population. Not much is known about sex differences in that regard.

**Objective:**

To explore sex-specific differences associated with the extent of BME in the SIJ suggestive of axSpA in a general population cohort study.

**Methods:**

Taking advantage of 793 recently evaluated MRIs of subjects < 45 years taking part in the SHIP cohort, we used negative-binomial (NB) count data regression to analyze factors associated with the extent of SIJ BME. Predictors were explored by model-based boosting (MBB), a machine learning approach.

**Results:**

Estimates of NB regression showed strong effects of sex in interaction with age, BMI, back pain, and particularly HLA-B27. The NB regression model showed incidence rate ratios (IRR) for the main effect of sex (females vs. males): 0.94 [95% CI: 0.63; 1.41], HLA-B27: 4.32 [2.09; 9.8], and for the interaction of sex to HLA-B27: 0.22 [0.06; 0.75]. According to MBB, HLA-B27 positivity, BMI, current smoking, back pain in the last 3 months, the interaction of sex and HLA-B27, and delivery in the last 12 months were of highest importance to explain the extent of SIJ BME.

**Conclusions:**

Different factors were associated with the extent of SIJ BME in females and males. Most importantly, HLA-B27 was relevant only in males but not in females in whom a postpartal state was important. This finding may be relevant for the pathogenesis of axSpA.

## Key messages

The main result of this cohort study is that the extent of lesions in the sacroiliac joints detected by MRI (bone marrow edema) in a young population < 45 years is influenced by HLA B27, an MHC class I allele which is strongly associated with axial spondyloarthritis—only in males. This suggests that sex-related differences matter in the pathogenesis of this disease, and as a possible clinical consequence, it possibly indicates that being HLA B27+ may have to be differently interpreted in females with back pain.

## Introduction

While ankylosing spondylitis (AS) had been widely recognized as a male disease [1], this is different in axial spondyloarthritis (axSpA), where the male/female ratio is more or less equal [2], and especially in patients with non-radiographic axSpA (nr-axSpA), where the proportion of women is even higher [3]. However, there is some evidence of significant differences between male and female patients with axSpA in clinical parameters including new bone formation [4–7], the persistence of inflammation [8, 9], and the response to anti-tumor-necrosis-factor (TNF) therapy [10].

Using data of 793 subjects aged <45 years participating in the general population based cohort Study [11] of Health in Pomerania (SHIP) who had undergone whole body magnetic resonance imaging (MRI [12]), we have recently reported that many individuals had minor MRI findings in the sacroiliac joints (SIJ) partly ressembling lesions detected in axSpA and the spine [13]. A second analysis, in which we examined factors associated with those MRI findings, showed that a history of previous delivery and HLA-B27 were important contributors to the occurrence and extent of such SIJ changes [14]. Thereafter, we decided to stratify for participants’ sex and found differences between males and females in the relevance of covariates for the extent of bone marrow edema (BME) in the SIJ. In the present study, we explored interactions of participants’ sex with all previously identified predictors of SIJ BME. To increase the credibility of the results, we compared those from conventional regression techniques with the results of a machine learning approach that were obtained after resampling.

## Methods

MRI imaging of SIJ was achieved using a 1.5-Tesla system (Magnetom Avanto; Siemens Medical Solutions, Erlangen, Germany) as part of a Whole-body MRI protocol [15]. Prior to the MRI examination all participants underwent an informed consent process. The present study analyzed coronary turbo inversion recovery magnitude (TIRM) sequences of the pelvis. TIRM imaging were performed using the following imaging parameters: time of repetition (TR), 4891 ms; time of echo (TE), 65 ms; flip angle (FA), 180°; matrix, 240 × 320; and bandwidth, 150 Hz/pixel with a voxel size of 2.1×1.6×5 mm. For this explorative study, we used the data of the same sample of individuals as in previous studies [13, 14], i.e., participants <45 years from the general population that had undergone MRI examination in SHIP. The methodology of SHIP and the procedures of MRI readings have been previously described [13, 14]. In the present analysis, we focus on interaction of participants’ sex and several predictors of BME in SIJ, based on the Berlin MRI score based on the presence and extent of inflammatory SIJ lesions [16].

To model the count data (extent of SIJ BME), we applied negative binomial regression. To avoid model overfitting or the generation of spurious effects [17], we examined the effects of participants’ sex modelled in interaction with different predictors in separated models. The following covariates were considered: age, HLA-B27 positivity, high-sensitive C-reactive protein (hsCRP > 0.5mg/dL), a physically demanding job, physical activity, BMI, the smoking status, backpain in last 3 months (yes/no), and in females: delivery in the last 12 months prior to the MRI examination and the number of children reported. The relevance of the interaction term was examined using likelihood ratio tests (model with main effects only vs. model with main effects and interaction term). In the models comprising age, the covariate age has been mean centered to define males of mean age as the reference in these models.

In addition, we applied model-based boosting (MBB), a machine learning (ML) approach, to compute the importance of variables for the prediction of the outcomes of interest. Similar to other ML techniques, boosting is designed to make accurate and robust predictions [18] which implies the use of resampling techniques. The main difference to conventional multiple regression is the model building approach that is done in a sequential manner to avoid overfitting [19]. In brief, starting from an intercept model, which means that no additional candidate variable is included in the model, each “base-learner” or candidate variable is evaluated in terms of fitting a loss function (negative log-likelihood). Only the best base-learner, with respect to minimizing residual sum of squares, is used to update the model in each iterative step [19]. In a model update, a shrunken model coefficient is included for the respective candidate variable. With each further iteration the respective model coefficient accrues if the candidate variable is selected again. The total number of iterations (*mstop*) determines the model complexity as more candidate variables can be included. Choosing the optimal *mstop* determines the model complexity which is conducted via resampling of the data and splitting them into training and test sets. The R package *mboost* [20] was used to build the MBB model in 1000 iterations. We included all previously found predictors as linear “base learners” as well as their interaction terms with participants’ sex. A 10-fold cross validation was applied to choose the optimal model (*mstop*). Variable importance of each base learner was calculated as the individual contribution to the model up to the optimal iteration number *mstop*. This measure is frequently used to interpret results of machine learning based applications [21].

Only few missing data were found in some covariates, mostly in HLA-B27 (*n*=37, 4.7%). The overall low frequency of missing values is presented in the results in detail. We therefore decided for imputation of single values only, also making use of the machine-learning approach. The R package *mice* was applied to impute missing data [22].

## Results

The characteristics of the study participants are shown in Table 1. Overall, 17.2% of the study population had BME in SIJ. No difference was found between females (16.2%) and males (18.1%, chi-square test *p*-value: 0.53). Similarly, regarding mean age, smoking status, and HLA-B27 positivity, no relevant differences were found related to sex. However, male participants were more often obese than females. Men also reported more physical activity and to have more physically demanding jobs. In women, elevated high-sensitive CRP levels were more frequently found than in males (10.2% vs. 3.1%, *p*-value: <0.01). The mean number of children in females was 1.4, and 16 women had delivered in the last year. Based on linkage with claims data [14], 9 participants may have had axSpA as suggested by the ICD-10 code M45.09 documented by a physician. However, 7 of these had no SIJ BME, which is similar to the prevalence of SIJ BME in the remaining population of this study.

**Table 1:** Characteristics of study participants

	Females	Males	All
***N***	401	392	793
**Age (years)**			
Mean (SD)	37.7 (6.03)	36.8 (6.54)	37.3 (6.29)
Median [Min, Max]	39.0 [21.0, 45.0]	38.0 [21.0, 45.0]	39.0 [21.0, 45.0]
**BMI categories acc. WHO**			
<25	227 (56.6%)	130 (33.2%)	357 (45.0%)
25–<30	108 (26.9%)	179 (45.7%)	287 (36.2%)
>=30	66 (16.5%)	83 (21.2%)	149 (18.8%)
**Physically demanding job**			
No	297 (74.1%)	213 (54.3%)	510 (64.3%)
Yes	104 (25.9%)	179 (45.7%)	283 (35.7%)
**Smoking status**			
Never	150 (37.4%)	145 (37.0%)	295 (37.2%)
Previous	133 (33.2%)	117 (29.8%)	250 (31.5%)
Current	118 (29.4%)	129 (32.9%)	247 (31.1%)
Missing	0 (0%)	1 (0.3%)	1 (0.1%)
**Back pain in last 3 month (Y/N)**			
No	154 (38.4%)	187 (47.7%)	341 (43.0%)
Yes	247 (61.6%)	204 (52.0%)	451 (56.9%)
Missing	0 (0%)	1 (0.3%)	1 (0.1%)
**SIJ: affected quadrants (Y/N)**			
Yes	65 (16.2%)	71 (18.1%)	136 (17.2%)
No	336 (83.8%)	321 (81.9%)	657 (82.8%)
**HLA-B27 positive**			
Yes	34 (8.5%)	33 (8.4%)	67 (8.4%)
No	351 (87.5%)	338 (86.2%)	689 (86.9%)
Missing	16 (4.0%)	21 (5.4%)	37 (4.7%)
**hsCRP > 0.5mg/dl**			
Yes	41 (10.2%)	12 (3.1%)	53 (6.7%)
No	345 (86.0%)	363 (92.6%)	708 (89.3%)
Missing	15 (3.7%)	17 (4.3%)	32 (4.0%)
**Average physical activity (annual)**			
>2h	73 (18.2%)	94 (24.0%)	167 (21.1%)
1–2h	262 (65.3%)	224 (57.1%)	486 (61.3%)
<1h	66 (16.5%)	74 (18.9%)	140 (17.7%)
**No. of children (women only)**			
Mean (SD)	1.37 (0.969)	na	0.692 (0.971)
Median [Min, Max]	1.00 [0, 7.00]	na	0 [0, 7.00]
**Birth within 1 year prior SHIP**			
Yes	16 (4.0%)	na	16 (2.0%)
No	385 (96.0%)	na	777 (98.0%)

The negative binomial regression analysis revealed differences between females and males regarding age, BMI, current smoking (vs. never), back pain, and HLA-B27 (Table 2), while the bivariate contingency Table 3 shows the frequency of SIJ BME in males and females stratified for HLA-B27 positivity.

**Table 2:** Estimates are presented as incidence rate ratios (IRR) for the outcome of BME in the SIJ which resulted from 10 negative binomial regression models. Models of type 1 contained the main effects only and models of type 2 also the interaction of participants’ sex

Effect	Model 1		Model 2		Likelihood ratio test^a^
	IRR	95% CI	IRR	95% CI	Model 1 vs. model 2 (*p* value)
Sex (females vs. males)	0.73	[0.49; 1.07]	0.74	[0.50; 1.09]	
Age (per decade, mean centered)	1.20	[0.88; 1.64]	1.48	[0.98; 2.25]	
Sex (females) to age (per decade)			0.62	[0.33; 1.15]	0.13
Sex (females vs. males)	0.80	[0.54; 1.18]	0.94	[0.63; 1.41]	
HLA-B27 (positive)	2.55	[1.44; 4.70]	4.32	[2.09; 9.80]	
Sex (females) to HLA-B27 (positive)			0.22	[0.06; 0.75]	0.02
Sex (females vs. males)	0.72	[0.48; 1.06]	0.72	[0.48; 1.08]	
hsCRP > 0.5mg/dL	1.16	[0.53; 2.57]	1.26	[0.29; 6.59]	
Sex (females) to hsCRP > 0.5mg/dL			0.89	[0.14; 4.99]	0.89
Sex (females vs. males)	0.74	[0.49; 1.09]	0.73	[0.44; 1.19]	
Physically demanding job	1.08	[0.72; 1.63]	1.07	[0.63; 1.83]	
Sex (females) to physically demanding job			1.03	[0.45; 2.38]	0.94
Sex (females vs. males)	0.73	[0.49; 1.08]	0.69	[0.28; 1.64]	
Phys. Activity (1–2h/w)	1.00	[0.61; 1.64]	0.95	[0.49; 1.83]	
Phys. Activity (<1h/w)	1.39	[0.76; 2.56]	1.40	[0.63; 3.13]	
Sex (females) to Phys. activity (1-2h/w)			1.12	[0.41; 3.08]	0.96
Sex (females) to Phys. activity (<1h/w)			0.99	[0.29; 3.43]	
Sex (females vs. males)	0.86	[0.58; 1.28]	0.92	[0.47; 1.78]	
BMI (25–<30 vs <25)	2.13	[1.36; 3.34]	2.32	[1.24; 4.42]	
BMI (≥30 vs <25)	1.69	[0.99; 2.90]	1.63	[0.75; 3.57]	
Sex (females) to BMI (25–<30 vs <25)			0.81	[0.33; 2.01]	0.82
Sex (females) to BMI (≥30 vs <25)			1.11	[0.37; 3.29]	
Sex (females vs. males)	0.76	[0.51; 1.12]	0.9	[0.47; 1.72]	
Smoking (previously vs. never)	0.90	[0.55; 1.46]	0.92	[0.46; 1.84]	
Smoking (current vs. never)	1.60	[1.02; 2.54]	1.94	[1.04; 3.63]	
Sex (females) to smoking (previously)			0.95	[0.36; 2.51]	0.63
Sex (females) to smoking (current)			0.66	[0.26; 1.64]	
Sex (females vs. males)	0.72	[0.49; 1.05]	1.05	[0.55; 1.99]	
Backpain last 3m (NRS)	1.75	[1.17; 2.62]	2.32	[1.34; 4.03]	
Sex (females) to backpain last 3m (NRS)			0.55	[0.24; 1.22]	0.14
Sex (females vs. males)	0.69	[0.38; 1.23]	0.68	[0.45; 1.00]	
Sex (females) to pregnancy (last 12m)			2.77	[0.88; 10.69]	0.08
Sex (females vs. males)	0.69	[0.38; 1.23]	0.69	[0.38; 1.23]	
Sex (females) to No. of children			1.04	[0.76; 1.42]	0.80

^a^Likelihood ratio test: each model was defined as (1) comprising main effects only and (2) including also the respective interaction term

**Table 3:** Bivariate contingency table of the frequency of SIJ BME in males and females stratified for HLA-B27 positivity

Sex	HLA-B27	SIJ BME count (Berlin score)			
		None	1	2	≥3
Male	negative	296	44	12	7
	positive	25	4	1	3
Female	negative	307	35	19	5
	positive	29	4	2	0

Regarding HLA-B27, the incidence rate ratio (IRR) with confidence intervals (CI) for females vs. males was 0.94 [0.63; 1.41], the main effect for HLA-B27 was 4.32 [2.09; 9.80], and for the interaction sex and HLA-B27 0.22 [0.06; 0.75]. Thus, HLA-B27 positivity was associated with the extent of SIJ BME only in males. A similar association was found for back pain: the IRR for females vs. males was 1.05 [0.55; 1.99], for back pain 2.32 [1.34; 4.03], and for the interaction 0.55 [0.24; 1.22]. In females, delivery within the last 12 months was strongly associated with the extent of SIJ BME (Table 2). No relevant interaction effects were found for hsCRP > 0.5mg/dL, physically demanding jobs, and physical activity.

**Fig. 1:**
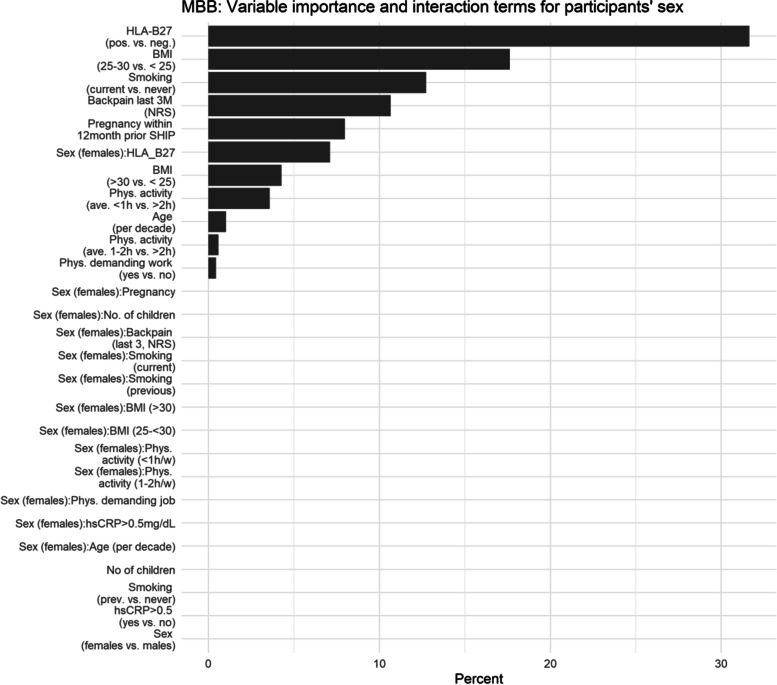
Variable importance for the prediction of the extent of BME in the SIJ (Berlin score) from a boosting model with implicit variable selection. This implies that all main effects and interaction terms with no filled bars have been removed in the optimal model. In mboost (23), categorical base learners are transformed to dummy-coded variables. Therefore, single levels of respective categorical variables may enter the model. Variable importance is measured as the summarized contribution of each base learner (linear effect of each base learner) to the final model. The final model is determined by 10-fold cross validation. The percentage (*x*-axis) of each variable quantifies the contribution of each variable to the optimal model determined by 10-fold cross-validation

The variable importance obtained by model-based boosting (MBB) showed similar results as the negative binomial regression with respect to the relevance of HLA-B27 (Fig. 1). The main effect of HLA-B27 and the interaction term with participants’ sex were included in the optimal model (mstop = 591 after cross validation). In addition, age, BMI, smoking (previous or current), backpain last 3 months as assessed by a numerical rating scale (NRS), physical activity, and a physically demanding job were associated with BME in the SIJ. In females, pregancy in the last 12 months was the most important predictor of BME.

## Discussion

The results of this explorative study suggest a remarkable differences of the covariate sex for the extent of BME in the SIJ of the general population. The main result, which is possibly related to the different pathogenesis of axSpA in men and women, is the different relevance of HLA-B27. Thus, being HLA-B27+ did matter for the extent of SIJ BME in males but not in females in our study. For women, a history of delivery in the last 12 months prior to the MRI examination and their BMI were more important to predict SIJ BME. As such, the question arises if these sex differences may present a novel insight into the pathogenesis of axSpA. The mechanic origin of enthesitis [23, 24], sacroiliitis, and spondylitis [25] has been frequently discussed but the link between acute and chronic inflammation and mechanic stress on the one and immunpathology and the strong genetic influence represented by HLA-B27 and other genes [26] on the other side is incompletely understood. Based on the results presented here, the influence of HLA-B27 on BME in SIJ is different in males and females. Before discussing this in more detail, we stress that the strong association of delivery with osteitis condensans ilii has now been confirmed on the population level—again, providing evidence for a strong link between physical stress and BME in the SIJ [13]. There is indeed now increasing evidence that BME frequently occurs in postpartal women already a few days after birth and also after 6, 12, and even > 24 months [27, 28].

On the background of a relatively high HLA-B27 prevalence of 8% in the population, a high prevalence of BME in the population [13], and a high proportion of axSpA patients also fulfilling criteria for fibromyalgia [29], and given that an HLA-B27 association was not present in female study participants, a possible conclusion could be that the presence of some BME in a HLA-B27+ obese female patient with back pain may finally not be a sufficient argument for a diagnosis of axSpA. Thus, the risk factors for BME in men and women seem different—one being HLA-B27. This is consistent with our view that this allele rather acts as a severity and not as a susceptibility factor [25].

It is well known that the disease course of patients with axSpA is different in males and females, and the proportion of males with new bone formation and ankylosis is higher than in females [7–10]. Accordingly, in the OASIS cohort, HLA-B27+ men but not women had a clearly higher progression than HLA-B27- men [30]. In the SPondyloArthritis Caught Early (SPACE) cohort, the presence of clinical SpA features was similar in male and female axSpA patients, but HLA-B27 and imaging were more often positive in men [31].

Along that line, in the nr-axSpA part of the spectrum when compared to radiographic axSpA, the proportion of females is always higher and HLA-B27 often less frequent [3]. This has led to intense discussions on whether nr-axSpA and r-axSpA are one or rather two diseases. However, it has also been intensively discussed that female axSpA patients may be undertreated [32].

In polygenic models of AS, female patients require a higher genetic load to develop disease and are thus more likely to have affected children [33]. In a recent study with 1178 members of the Swiss AS Patient Society diagnosed with axSpA that started as early as in 1985, male and female patients with AS were found to have a genetically similar disease, and men with nr-axSpA had a polygenic risk score [34] very similar to male AS patients. The latter was also distinct from healthy subjects, but women with nr-axSpA had similar polygenic risk scores to healthy subjects and were, on the other hand, distinct from female AS patients [35, 36].

As recently explained [33], polygenic disorders with each gene following Mendelian rules may lead to polygenic trait distributions, and only individuals with more than a threshold level of susceptibility would develop the disease concerned. This widely accepted theory underpins modern genetic statistical methods assessing disease heritability in dichotomous traits. The gender with the lower prevalence requires a higher genetic risk before it develops the disease. Thus, overall, women with AS should have a higher genetic risk than men with the disease [26].

In addition, the frequency of IL-17A and Th17 cells, key factors in the inflammatory Th17 axis, was elevated in male patients with AS but not in female patients with AS [34]. Male and female patients with AS displayed shared gene expression patterns, but male AS patients had additional alterations in gene expression that were not seen in female patients with AS. The differential sex-related immune profiles were independent of HLA-B27 status, clinical disease activity, or treatment implicating intrinsic sexual dimorphism in AS [37].

Finally, it is worth mentioning that smoking and age came out as predictive factors in men but not in women. The significance of smoking has been discussed for patients with axSpA and there is some evidence that it has negative impact on radiographic progression in axSpA [38]. However, this is a population-based study. A negative effect of smoking on low back pain has been reported [39]. Little is known about sex differences in this regard.

An influence of age on alterations of the SIJ has been described in studies using computed tomography, the gold standard to detect degeneration. Major differences between men and women regarding alterations of the SIJ have also been found in this study [40].

A limitation of this explorative study is the selected sample of the SHIP cohort with volunteers from the general population [11]. However, the total sample size of 793 MR images gave a unique oppurtunity to conduct these analyses—even though, the numbers of MRI findings in subgroups were expectedly low. Regression models had to be parallelized to avoid overfitted models but hereby the chance of false positive findings was increased due to multiple testing. Therefore, resampling techniques were applied to mitigate these sources of bias and the method affirmed major findings of regression techniques. However, the handling of missing values in the machine-learning approach was restricted to the use of single imputations only. Therefore, a replication of these results in other cohorts and further data are required to confirm these study results.

## Conclusions

In conclusion, although an almost similar distribution of SIJ BME was seen in males and females, we found remarkable differences in the role of covariates driving the extent of MRI lesions in the SIJ between male and female subjects in a large population-based cohort. Thus, positivity of HLA-B27 plays an important role mainly in men while in females a different factor, the postpartal state, is more relevant. Consistent with the results of recent genetic studies this finding has clinical impact because it suggests that a positive HLA-B27 result may have to be interpreted differently in female patients under suspicion of axSpA. However, this clearly needs further study.

## Data Availability

Supporting data are available.

## Abbreviations

axSpA: Axial spondyloarthritis
r-axSpA: Radiographic axSpA
nr-axSpA: Non-radiographic axSpA
MRI: Magnetic resonance imaging
TIRM: Turbo inversion recovery magnitude (MRI sequence)
BME: Bone marrow edema
SIJ: Sacroiliac joints
HLA-B27: Human leucocyte antigen B27 (MHC class I)
hs-CRP: High-sensitive C-reactive protein
TNF: Tumor-necrosis-factor
IRR: Incidence rate ratio
CI: Confidence interval
NB: Negative-binomial (count data regression)
MBB: Model-based boosting (a machine learning approach)
SPACE: SPondyloArthritis Caught Early cohort
SHIP: Study of Health in Pomerania
